# NK Cell Hyporesponsiveness: More Is Not Always Better

**DOI:** 10.3390/ijms20184514

**Published:** 2019-09-12

**Authors:** Marie Frutoso, Erwan Mortier

**Affiliations:** 1 CRCINA, CNRS, Inserm, University of Nantes, F-44200 Nantes, France; 2 LabEX IGO, Immuno-Onco-Greffe, Nantes, France

**Keywords:** unresponsive, exhaustion, cytokines, cancer, virus, interleukin

## Abstract

Natural Killer (NK) cells are a type of cytotoxic lymphocytes that play an important role in the innate immune system. They are of particular interest for their role in elimination of intracellular pathogens, viral infection and tumor cells. As such, numerous strategies are being investigated in order to potentiate their functions. One of these techniques aims at promoting the function of their activating receptors. However, different observations have revealed that providing activation signals could actually be counterproductive and lead to NK cells’ hyporesponsiveness. This phenomenon can occur during the NK cell education process, under pathological conditions, but also after treatment with different agents, including cytokines, that are promising tools to boost NK cell function. In this review, we aim to highlight the different circumstances where NK cells become hyporesponsive and the methods that could be used to restore their functionality.

## 1. Introduction

The development of strategies to cure cancer and infection has captivated scientists for centuries. Among these strategies, immunotherapies have provided the most significant advance in cancer therapy in the last 30 years. A wide array of immune cell types contribute to immunosurveillance and Natural Killer (NK) cells are of particular interest for the critical role they play in antitumor host defense. They were first discovered in 1975 for their ability to kill target cells without prior immunization [1,2]. In addition to their spontaneous cytotoxicity, NK cells are able to produce cytokines and chemokines that activate local immune cells and recruit additional immune cells, conferring upon them an important role at the interface between innate and adaptive immunity [3]. Furthermore, while NK cells were long considered as short-lived effector cells, more recent studies have identified long-lived effector NK cells possessing a memory phenotype [4,5,6]. These properties highlight the great potential of NK cells for use in immunotherapy, making it clear why there are efforts to harness their function and increase their activation status.

NK cell biology is controlled by a vast array of cytokines such as the interleukins (IL)-2, -12, -15 or -21. IL-12 has been shown to be important for NK cell activation [7], and IL-21 participates in NK cell maturation [8]. IL-2 and IL-15 have shared functions including promoting the generation of cytotoxic T lymphocytes and NK cells and enhancing their proliferation. The primary cytokine involved in NK cell biology is IL-15 and the requirement of IL-15 in NK cell development is illustrated by their total absence in IL-15 or IL-15 receptor alpha chain (IL-15Rα) knock-out (KO) mice [9,10,11]. IL-15 is also involved in NK cell maturation [12] and activation [5,13,14,15,16]. Type I interferons (IFN) have also be shown to influence NK cell homeostasis and function [17]. However, this is an indirect effect as the impact of IFN was linked to the release of IL-15 by dendritic cells which, in turn, promoted the survival of NK cells [18,19], therefore confirming the predominant role of IL-15 in NK cell biology.

NK cell activation is tightly regulated by a family of activating and inhibitory receptors. Indeed, NK cells have to integrate signals from multiple receptors in order to sense their environment and respond appropriately [20,21]. The most studied inhibitory receptors are those which recognized the Human Leucocyte Antigens (HLA) [22]. They are associated with an Immunoreceptor Tyrosine based Inhibitory Motif (ITIM)-bearing molecule that transmits inhibitory signals preventing cytotoxicity when the ligand is encountered. The inhibitory receptors consist of the C-type lectin family (with receptors such as NKG2A and KLRG1), the Killer-cell Immunoglobulin-like Receptor (KIR) family in humans [20] and the Ly49 family in mice [23]. To mount a productive response, a critical threshold of activating signaling that exceeds the counterbalancing influence of the inhibitory receptors must be achieved. The dominant activating receptors are associated with an Immunoreceptor Tyrosine based Activation Motif (ITAM)-bearing molecule, such as the three natural cytotoxicity receptors (NCR), NKp46, NKp30 and NKp44. There are other activating receptors such as NKG2D which is associated with the signaling molecule DAP10, or costimulatory receptors such as 2B4, DNAM-1, CD2 and NKp80 [24,25,26]. Once activated, NK cells release the content of their lytic granules (containing perforin and granzyme B) to achieve cytotoxicity [27]. However, NK cells can also eliminate target cells through several methods: the death-receptor pathways involving FasL and TRAIL [28]; the secretion of pro-inflammatory cytokines and chemokines IFNγ, TNF, IL-6 GM-CSF and CCL5 [29,30]; or the engagement of the low-affinity activating receptor FcγRIIIa (CD16) that binds the Fc portion of immunoglobulin G1 (IgG1) and mediates antibody-dependent cellular cytotoxicity (ADCC) [31,32].

Given the increasing interest in harnessing the cytolytic potential of NK cells in cell therapy against cancer, different strategies are currently being investigated. The global aim of these strategies is to use drugs that will increase NK cell activation status either by blocking the expression of inhibitory molecules, and/or promoting the function of activating receptors through the upregulation of their related stress-induced ligands, and/or through the administration of cytokines known to potentiate NK cell response [33,34,35]. However, in the context of tumors and chronic infections, NK cells exhibit an exhausted phenotype similar to exhausted T cells [36], displaying poor effector functions and an altered phenotype [37]. Although the exact mechanisms leading to NK cell exhaustion in tumors and chronic infections are poorly defined, emerging studies have shown that multiple negative regulatory pathways in these contexts might contribute to the exhausted status of NK cells [38,39]. However, it has also been proposed that stimulation with activating signals or pro-inflammatory cytokines could also lead to an alteration of NK cell response. It is therefore important to be aware of the signals driving NK hyporesponsiveness. This review aims to offer a non-exhaustive overview of treatments and environments that alter NK cell function and the different strategies that could be used in order to overcome this defect.

## 2. NK Cell Hyporesponsiveness during the Education Process

### 2.1. Generalities on NK Cell Education

NK cells possess a high phenotypic diversity comprising up to 100,000 unique subsets in healthy individuals [40]. This diversity is mostly based on the random combination of germline-encoded activating and inhibitory receptors that bind to major histocompatibility complex (MHC) molecules and adjust NK cell function in a process called NK cell education [41,42]. This random combination could easily increase NK cell potential towards autoreactivity. Yet, there is no evidence that NK cells alone are able to cause autoimmunity. Thus, NK cell education and tolerance appear to be more effective than either the central or peripheral tolerance mechanisms governing T cells and B cells. To avoid autoreactivity of NK cells lacking inhibitory receptors that bind to MHC class I molecules of the host [43,44], or expressing activating receptors that recognize self-ligands including MHC molecules [22,45,46], an education system exists whereby such NK cells acquire self-tolerance. The potentially autoreactive NK cells are not generally clonally deleted but instead acquire a state of hyporesponsiveness to stimulation.

Two models have been proposed to account for the differential responsiveness of NK cells [47,48]. One “arming” model suggests that NK cells without inhibitory receptors simply fail to acquire full reactivity [44,47,48], while the “disarming” model predicts that NK cells without inhibitory receptors for self MHC I are rendered hyporesponsive due to chronic low-level stimulation [47,49]. Although there is evidence to support both models separately, it is also possible they work in tandem. Indeed, NK cell responsiveness has been described as a rheostat model: a continuous process where the availability of receptor ligands encountered in the normal environment tunes NK cell response to an optimal set point [50]. The following subsections give more details on NK cell hyporesponsiveness in the two different models of education.

### 2.2. NK Cell Hyporesponsiveness in the “Arming” Model

**Figure 1:**
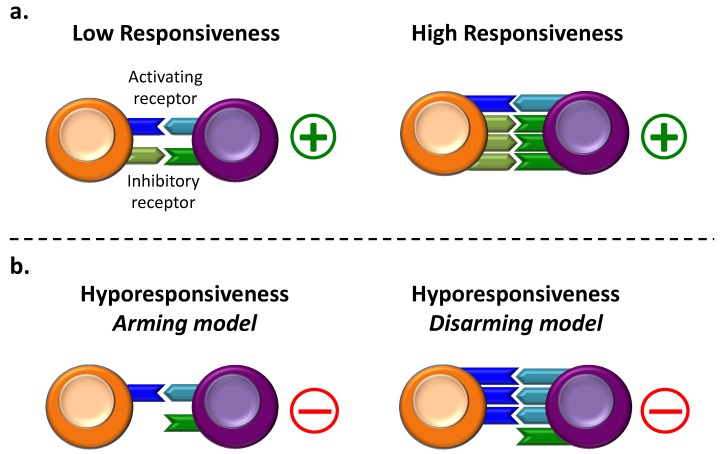
Natural Killer (NK) cell hyporesponsiveness during the education process. (**a**) Left. NK cells acquire functional maturation during development. The appropriate integration of inhibitory and activating signals determines the outcome of interactions to target cells. Right. High responsiveness (green plus) of NK cells after encountering several inhibitory receptor ligands during development. (**b**) NK cell hyporesponsiveness (red minus) could occur by (left) the absence of inhibitory signals or (right) an increased activating stimulation.

Inhibitory receptors are particularly important in terms of NK-cell education and their ability to distinguish self from non-self [51]. Indeed, the detection of damaged cells caused by viral infections or cancer could occur as a result of the absence or downregulation of HLA molecules [52,53] and NK cell hyporesponsiveness has been seen in the absence of MHC class-I specific inhibition. A subset of NK cells lacking all self-MHC class-I specific inhibitory receptors, which otherwise developed normally, was partially or even completely defective in killing MHC class-I deficient target cells in mice and humans [43,44,54,55]. The first report of NK cell activity in mice deficient for the Beta-2 microglobulin (β2m), a component of MHC class I molecules, demonstrated that NK cells in these mice were unresponsive to MHC class-I deficient and allogenic targets [56,57,58]. In other mouse models, NK cell responsiveness has been shown to be increased with each inhibitory receptor that can find its ligand during maturation [59,60]. Since the “arming” model proposes that functional maturation of NK cell precursors requires interaction with cognate MHC class-I positive cells [47,48], NK cells that do not encounter cognate MHC class-I molecules remain in an unarmed and therefore hyporesponsive state (Figure 1).

### 2.3. NK Cell Hyporesponsiveness in the “Disarming” Model

Activating receptors are responsible for the recognition and killing of infected or tumor cells. However, sustained engagement of these activating receptors could also induce NK cell hyporesponsiveness. The “disarming” model suggests that developing and mature NK cells are exposed to persistent stimulation delivered by normal cells. In this scenario, NK cell hyporesponsiveness occurs when normal cells stimulate NK cells and concomitantly fail to provide inhibitory signals via MHC class-I molecules, or when excessive stimulation overcomes inhibitory signals. Continuous engagement of the activating receptor results, surprisingly, in the hyporesponsiveness of murine and human NK cells [30,61,62,63]. For instance, chronic engagement of NKG2D in a transgenic mouse model of ubiquitous NKG2D ligand expression (H2-Kb-MICA mice) impacts NK cell responsiveness, affecting Ly49D mediated cytotoxicity and IFNγ secretion [64]. Sustained engagement of NKG2D by MICA was shown to cause receptor internalization and degradation in vitro [62,65]. Accordingly, the transgenic expression of Rae-1, another NKG2D ligand, also results in a reduced expression of the NKG2D receptor and impairment of NKG2D-mediated NK cell function [30,61]. In addition, chronic exposure to m157, the ligand for the Ly49H activating receptor, decreases the expression of Ly49H receptor and leads to impaired NK cell activation [66,67,68]. This NK cell hyporesponsiveness could be due to the acquisition of activation receptor ligands, such as m157, by trogocytosis [69]. The dynamic distribution of these receptors at the cell surface also participates in the education of NK cells [70]. Indeed, it has been shown that NKp46 diffuse slowly on the surface of hyporesponsive cells compared to responsive NK cells that possess self-specific inhibitory receptors. In contrast, the inhibitory receptor Ly49A was more constrained and diffused more slowly on responsive cells [71]. It is now well appreciated that continuous engagement of the Ly49H activating receptor on NK cells results in hyporesponsiveness of the NK cells due to altered signaling pathways downstream of the receptor and adaptor molecule [72]. This phenomenon of NK cell hyporesponsiveness has also been observed in humans where continuous engagement of the activating receptor KIR2DS1 impairs the responsiveness of NK cells [30]. Thus, in the absence of efficient inhibitory signals, NK cells experience sustained activation and become hyporesponsive, which is known as the “disarming” model [47] (Figure 1).

## 3. NK Cell Hyporesponsiveness Following Activating Stimuli

### 3.1. NK Cell Hyporesponsiveness in Cancer

#### 3.1.1. First Observations: Exposure with Target Cells

**Figure 2:**
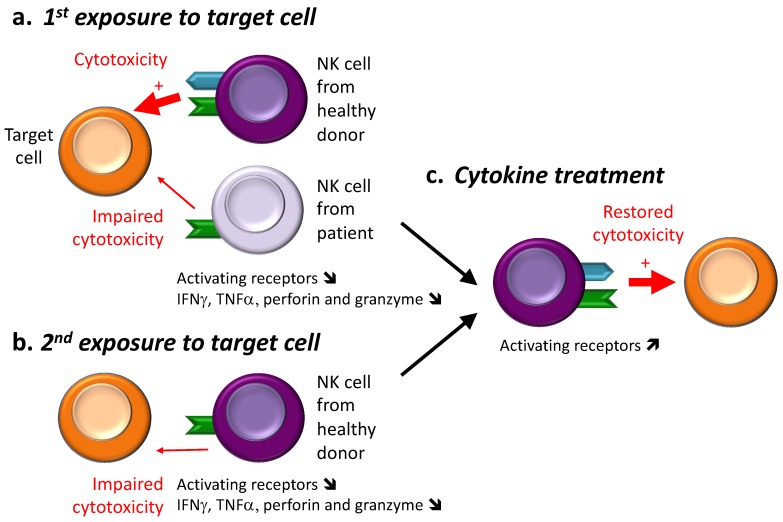
NK cell hyporesponsiveness in tumor or infection contexts. (**a**) When tested in vitro for their cytolytic function, NK cells from healthy donors are able to kill virally infected or tumor cells (red bold arrow). In contrast, peripheral blood NK cells from virally infected patients or intratumoral NK cells from tumor-bearing patients have lower expression of activating receptors and a decreased ability to degranulate and to produce IFNγ or TNFα (impaired cytotoxicity, red unbold arrow). (**b**) During a second exposure to target cells, NK cells from healthy donors, become hyporesponsive with shared features with NK cells from virally infected or tumor-bearing patients (i.e., decreased expression of activating receptor, decreased degranulation and decreased production of IFNγ, TNFα). (**c**) Treatments with cytokines have been shown to restore NK cell functionality in both cases.

Cancer progression is described as a process called immunoediting that has 3 phases, the 3E: Elimination, Equilibrium and Escape [73]. Throughout this process, tumor cells gain immunosuppressive properties while immune cells progressively lose their anti-tumor activity and reach an exhausted phenotype, which in turn, favors tumor growth. This exhausted phenotype arises during many chronic infections and cancers and was first highlighted in the 1980′s during in vitro cytotoxic experiments. It has been described that NK cells previously exposed to target cells, lose their lytic activity during a second exposure to the same or even other target cells [74]. The induction of NK cell hyporesponsiveness was dependent on cell-cell contact, however these NK cells were still capable of forming conjugates. This hyporesponsiveness was characterized by downregulation of perforin and granzyme mRNA [75], weak PKC translocation [76], and no phosphatidyl inositol turnover [77]. Thus, while the first exposure results in the killing of the tumor cell, it also induces NK cell hyporesponsiveness rendering them unable to exert their cytotoxic function and suggesting a tumor-induced local impairment of NK cells (Figure 2).

#### 3.1.2. Overexposure to Ligands of Activating Receptors

Interestingly, tumor cells can upregulate NK cell activating receptor ligands, inducing exhaustion and hyporesponsiveness instead of activation. For instance, the NKG2D ligand, MICA/B, was frequently expressed in solid tumor [78] and leukemia [79] and soluble MICA/B downregulates NKG2D expression, resulting in decreased killing potency [65]. The localized chronic exposure to tumor cells expressing NKG2D ligand alters NKG2D signaling and participates in tumor evasion from NK cells [61,80]. Other activating receptors, including NKp46, NKp30, DNAM-1, 2B4 and CD94/NKG2C, were also downregulated on NK cells from patients suffering from acute myeloid leukemia (AML), in which NK cells have also been shown to have decreased production of TNFα, IFNγ, perforin and granzymes compared to age-matched controls [81]. Nectin-2 was also detected in numerous cancers [79], leading to downregulation of DNAM-1 on NK cells [82]. These observations further demonstrate that chronic exposure to activating receptors leads to NK cell hyporesponsiveness.

### 3.2. NK Cell Hyporesponsiveness and Viral Infections

Persistent chronic viral infections constitute a major health burden worldwide and afflict more that 500 million people. Persistent viruses impair overall immunity, including NK cells. The underlying mechanisms leading to NK cell hyporesponse are, however, not fully understood.

#### 3.2.1. HIV-1

Numerous studies have pointed out that Human Immunodefiency Virus 1 (HIV-1) infection impairs NK cell homeostasis and their antiviral effector functions. Indeed, frequencies, phenotype and function of NK cells are modified with a pathological redistribution of NK cell subsets, which results in an increase of the anergic CD56^-^ and a decrease of the cytotoxic CD56^dim^ fractions [83,84,85,86]. Phenotypical defects are also characterized by decreased surface expression of natural cytotoxic receptors such as NKp46, NKp30 and NKp44 and other activating receptors such as 2B4 [87]. Simultaneously, expression of inhibitory receptors as KIR2DL2 or LIR1/ILT2 have been shown to be increased [88,89,90]. Moreover, human NK cells from HIV-positive patients have reduced IFNγ production following in vitro IL-12 + IL-18 stimulation and a decreased perforin expression leading to an impaired specific lysis of the K562 tumor cell line [88,90,91]. Impairment of the cytolytic machinery of NK cells from HIV-infected patients also results in a failure to recognize and kill *Cryptococcus neoformans* (*C. neoformans*), a pathogenic yeast which is a leading cause of meningitis in Acquired Immune Deficiency Syndrome (AIDS) patients [92]. Taken together, these phenotypic and functional defects impair the overall NK cell antiviral, antimicrobial and antitumor activity in HIV-infected patients (Figure 2).

#### 3.2.2. Other Viruses

Other viruses, including herpes simplex viruses (HSV), influenza virus, hepatitis C virus (HCV) and cytomegalovirus (CMV), have been shown to impair NK cell function. In HSV-infected patients, prolonged exposure to the virus has been shown to alter NK cell function. Indeed, NK cells from HSV+ donors with recurring lesions have a lower activity as shown by a reduced degranulation response, which is not caused by a decreased recognition of the tumor target, as both coculture with K562 and PMA/ionomycin stimulation led to decreased degranulation [93,94]. It is, however, unclear whether the low NK cell degranulation in HSV+ patients is a consequence of ongoing viral reactivation or a primary event predisposing individual to relapse. An early study showed that NK cells can lose their cytotoxicity upon an 8-hour cell contact with HSV-infected targets [95] showing that NK cell inactivation could be an early event that affects the overall immune surveillance.

Patients with severe influenza infection were shown to have diminished NK cell numbers in peripheral blood with a decreased fraction of the CD56^dim^ population and an almost complete absence of pulmonary NK cells [96,97]. Decreased NK cell activity was also demonstrated in influenza virus-infected mice with a decreased natural cytotoxicity and a decreased generation of pro-inflammatory cytokines such as IFNγ or GM-CSF and chemokines such as MIP-1α, MIP1β or RANTES [98,99,100]. Furthermore, it has been shown that NK cells can be directly infected by the virus which causes the downregulation of the NKp46 associated-ζ chain through the lysosomal pathway leading to a decreased cytotoxic pathway mediated by NKp46 and NKp30 [100,101].

In the case of HCV infection, the frequency of NK cells in HCV+ patients has been shown to be decreased with a marked reduction in the CD56^dim^ cell fraction and an increase in the CD56^bright^ fraction [85]. NK cell exposure to HCV in vitro impaired NK cell functionality with the CD56^dim^ subset presenting reduced expression of activating receptors NKG2D, NKp46 or NKp30, a decreased production of IFNγ, and a decreased capacity to degranulate and lyse target cells [102,103]. Additionally, a role for the HCV serine protease NS3 could be at stake in NK cell impairment [103].

In murine CMV (MCMV) infection, NK cells recognized infected cells with the activating receptor Ly49H, which specifically interacts with the MCMV-encoded class I like protein m157 on virally infected cells [104,105]. It has been shown that mature wild-type NK cells adoptively transferred into transgenic C57Bl/6 mice that ubiquitously express m157 (m157-Tg) acquire hyporesponsiveness by 24 h, which is sustained at 72 h and 9 days post-transfer. This is evidenced by decreased Ly49H expression and a defect in IFNγ production upon ex vivo stimulation with plate-bound anti-NK1.1 [63,67,68]. These results indicate that continuous activating receptor engagement can result in NK cell functional defect.

#### 3.2.3. Virus and Cancer

Many cancers have well-known association with AIDS, mainly because of coinfection with oncogenic viruses such as Human Herpesvirus 8 (HHV-8) and Human Papilloma Virus (HPV). Indeed, coinfection of HIV with HHV-8 can lead to the formation of Kaposi Sarcoma (KS), and coinfection of HIV with HPV is associated with an increased risk of cervical cancer. NK cells from patients with KS have been reported to possess decreased activity [91] and to be hyporesponsive ex vivo following direct triggering of their activating receptors or short stimulation with NK cell targets [106]. The exact mechanism leading to NK cell hyporesponsiveness is unclear, however Beldi-Ferchiou et al. have linked it to an increased expression of PD-1 in a sub-population of activated, mature CD56^dim^ CD16^+^ NK cells [106]. Furthermore, this NK cell hyporesponsiveness was not different in HIV-positive or HIV-negative subjects, showing that HHV-8 is likely responsible for this impairment. Further studies have found that healthy carriers of HHV-8 (i.e., without developing KS and being HIV-negative) possess alterations in the NK cell receptor repertoire, indicated by reduced expression of activating receptors NKp46, NKp30 and CD161. These changes could impair NK functionality and preclude the efficient prevention and immunosurveillance of KS development [107]. In the same manner, NK cell defects were established in HPV-infected women with decreased ex vivo cytotoxicity [108], a reduction of different innate immune receptors including Toll-like receptor 9 (TLR9) [109], but also NKp30, NKp46 and NKG2D [110,111] which in turn impair NK cell response and clearance of the virus.

Finally, NK cell hyporesponsiveness triggered by viruses or tumor cells remains a poorly understood process. Indeed, even though NK cell modifications following virus or tumor cell exposure are quite similar, with a reduced CD56^dim^ fraction, a reduced expression of activating receptor and a decreased cytolytic ability, the reason of this phenomenon is unclear. Indeed, whether NK cell hyporesponsiveness is an early or a late event is uncertain. In vitro studies using healthy NK cells showed that NK cell exposure to infected-target or tumor cells is sufficient to decrease NK cell cytotoxicity [74,76,77,95,100,101,103]. However, human NK cells from the peripheral blood of infected patients or intratumoral NK cells from tumor-bearing patients possess the same altered cytolytic activity while experiencing a prolonged exposure with the virus, making us curious about the required duration of the signal to obtain NK cell hyporesponsiveness.

### 3.3. Recovery from NK Cell Hyporesponsiveness

Whether NK cell hyporesponsiveness arose after a short or a prolonged exposure to either cancer- or infection-related stimuli, numerous studies aim at restoring NK cell activity to increase the clearance of intracellular pathogens, viral infection and tumor cells. Several strategies are being used and consist of either modifying the surrounding NK cell environment through different blocking approaches of the immunosuppressive cytokines and regulatory cells; or directly restoring NK cell exhaustion through blockade of inhibitory receptors (KIR blockade, anti-NKG2A, etc.), blockade of inhibitory checkpoints (PD-1, CTLA-4), or upregulation of activating receptors such as NKG2D. The different approaches that could be used to restore/improve NK cell functionality in cancer/infection have been well-documented recently [34,35,37,39,112]. In the present review, we will focus on the key cytokines that have been shown to hold great promises in restoring NK cell hyporesponsiveness in cancer and infection.

#### 3.3.1. Cancer

Cytokine infusion mainly consists of the administration of cytokines known to induce activation and proliferation of NK cells. Among these cytokines, IL-2 (proleukin) and IFNα (Roferon-A) are already approved by the FDA for the treatment of renal carcinomas and metastatic melanomas, or for hepatitis C, hairy cell leukemia and AIDS-related Kaposi’s sarcoma, respectively. Indeed, their ability to mediate tumor regression [113,114] through their role in the activation of the immune system [115,116] is a useful way to limit cancer growth. However, as monotherapy, both cytokines are insufficient to improve patients’ survival and strategies to increase their efficacy are under investigation [117,118,119]. Moreover, as NK cells have been shown to be impaired after short or prolonged exposure to target cells, IL-2 and IFNα were also investigated for their potential to restore NK cell cytolytic activity. To that end, human NK cells from healthy donors that had impaired activity during a second in vitro exposure to tumor cell lines, had restored function following treatment with IL-2 [120,121]. Furthermore, the decreased cytolytic activity against tumor cell lines of intratumoral NK cells from cancer patients has also been shown to be increased/restored when IL-2 or IFNα are used either in pretreatment or during cytotoxic assays in vitro, showing that both cytokines could help in re-establishing NK cell function after a prolonged exposure to tumor cells [122,123,124,125,126] (Figure 2). However, for patients with myelodysplastic syndrome (which is a precursor to AML), IL-2 treatment seems to be inefficient in restoring NK cell responsiveness. Indeed, while culture with IL-2 results in an upregulation of NKp46 on NK cells, their cytolytic activity remains deeply altered [127]. IL-2 ability to reverse NK cell anergy was also shown in vivo in RMA-S tumor bearing mice. The mechanism behind IL-2 reversing NK cell anergy is, however, unclear. Indeed, studies are contradictory about the correct timing of treatment. In some studies, pretreatment was useless for restoring NK cell activity [80,120], and other studies have shown that treatment during tumor progression or after separation of NK cells from the tumor was able to reverse the NK cell anergic state [80,128].

Other cytokines involved in NK cell development have also been investigated. IL-12, for example, which is an important factor for NK cell activation [7,129] was shown to be a promising immunotherapeutic agent [130]. The combination of IL-12 with IL-18 is particularly attractive as it leads to a population of memory-like NK cells that further proliferate in mice and in humans when re-exposed to IL-12 + IL-15 [5,16,131]. In that line, IL-12 in combination with IL-18, strengthens NK cell cytotoxicity, and has also been shown to be an efficient treatment to restore NK cell activity in RMA-S tumor bearing mice [128].

Another cytokine of interest for immunotherapy is IL-15. Indeed, IL-15, which belongs to the IL-2 family, is able to stimulate both the innate and adaptive systems and is the most crucial cytokine involved in the development, maturation, and activation of NK cells [9,10,11]. In addition to these properties, IL-15 has no marked effect on regulatory T cells and it inhibits the activation induced cell death by IL-2 [132,133]. Since 2008, IL-15 has become one of the most promising molecules in antitumor therapy [134]. IL-15 is also an interesting cytokine to restore human NK cell functionality in cancer. Indeed, culture of human NK cells from AML patients or Ewing sarcoma patients with IL-15 restores their expression of activating receptors and their cytotoxicity against target cell lines [135,136]. In another context, after an initial lysis of melanoma cell lines by IL-2-activated human NK cells from healthy donors, melanoma cells acquired resistance to these IL-2-activated cells preventing cell lysis. IL-15 treatment partially overcame this tumor escape mechanism, indicating that different successive treatments are required to restore NK cell response and effectively fight tumor cells [137].

Finally, in the context of cancer, the mechanism leading to a reversal of NK cell hyporesponsiveness by one of these four cytokines remains unclear. Indeed, most of the studies have only considered cytolytic activity and have shown that using one or more of these cytokines helps to restore a “baseline” level of activity [120,121,122,123,128] (Figure 2)**.** Three studies have pointed out a more precise role for these cytokines in restoring the expression of activating receptors such as NKG2D or DNAM-1 that lead in turn to a restored cytotoxic activity [80,135,136].

#### 3.3.2. Infections

In a cancer-related environment, reversing NK cell hyporesponsiveness during chronic infection has been of interest and some cytokines have been shown to restore NK cell function. IFN has been shown to increase the frequency of CD56^dim^ NK cells and to restore NK cell activity in chronic hepatitis C patients, which could reduce the risk of hepatocarcinogenesis [138]. Administration of TNFα, IFNβ and IL-12 [68] or IL-2 [63] was able to restore NK cell responsiveness in a transgenic mouse model where a continuous engagement of Ly49H and m157 impairs NK cell activity. However, while this continuous engagement mimics an MCMV infection, the authors demonstrated that NK cells were not hyporesponsive following acute MCMV challenge due to a downregulation of MHC class-I molecules and an inflammatory environment. These phenomenon do not occur during prolonged engagement between m157 and Ly49H in the transgenic model, showing that cytokines were able to restore NK cell responsiveness in an MHC class-I independent mechanism [68].

Antiretroviral therapy (ART) is the current combination of drugs used to restrain HIV-1. ART has been shown to restore many aspects of NK cell immunity such as restoring 2B4 expression and reducing the activity of NK inhibitory receptor [87,89] but some critical functions of NK cells remain compromised including low levels of NK activating receptors [89], pointing to the requirement of other drugs to reestablish NK cell functions. IL-15 could be one of these molecules as treatment of PBMC from HIV-infected patients with this cytokine (but not IL-2) has been shown to restore NK cell cytotoxic activity [83,84,85,139]. As for NK cells from HIV-infected patients, IL-15 treatment (but not IL-2) has been shown to restore NK cell degranulation and IFNγ production from PD-1^+^ NK cells from Kaposi sarcoma-bearing patients [106]. Furthermore, despite ART, 1 million cases of crytptococcal meningitis occur annually worldwide [140]. IL-12 has been proven to restore NK cell anticryptococcal killing from HIV-infected patients at least partially through restoration of NKp30 expression [92,141] (Figure 2).

## 4. NK Cell Hyporesponsiveness Emergence after Treatment

Finally, treatments with IL-2, IL-12, IL-15 and IFNα have been proven to restore NK cell functionality after a short or a prolonged exposure to a stimulus. This action could help in preventing the acceleration of the 3E process (or infection) and insure a better NK cell functionality during the escape process. In regard to their anti-tumor potential, these cytokines have been extensively studied in order to increase NK cell activity and improve the clearance of tumor or infected cells before the escape phase characterized by an over-suppressive environment.

### 4.1. Cytokine Treatments and NK Cell Hyporesponsiveness

#### 4.1.1. In Mice

Since the 1980′s, impact of treatments with IFNα, IL-2, IL-12 or IL-15, on NK cells have been examined in mice. Unexpectedly, while a single treatment with one of the above-mentioned cytokines leads to an increased NK cell cytotoxic activity, multiple treatments with one cytokine was shown to be negatively correlated with NK cell cytotoxic activity [142,143,144,145,146,147,148]. This NK cell hyporesponsiveness driven by cytokines was shown to be systemic [144,148], indicating that the origin of this phenomenon was not linked to a redistribution to other organs.

**Figure 3:**
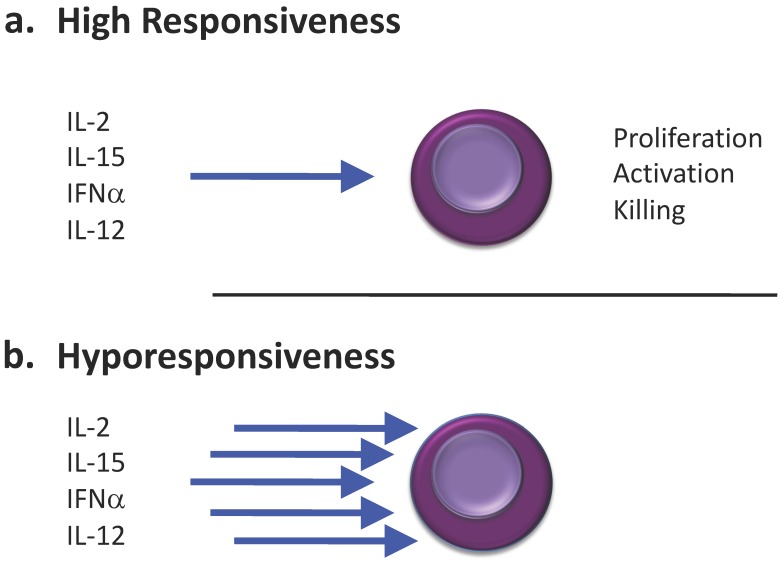
NK cell hyporesponsiveness after treatments. (**a**) NK cell functions could be increased by cytokine stimulation (blue arrows). (**b**) Repeated administration or sustained activation could result in NK cell hyporesponsiveness.

Moreover, regarding the immunotherapeutic potential of IL-15 for the treatment of cancers, several groups have developed IL-15 agonists mostly based on the IL-15/IL-15Rα complex. These agonists possess an increased efficiency compared to IL-15 in their ability to activate NK cells [149,150,151,152]. However, similar to IL-15, repeated treatments with IL-15 agonists lead to NK cell hyporesponsiveness. This NK cell hyporesponsiveness following multiple treatments is systemic and characterized by impaired activation (altered balance of activating and inhibitory receptors, decreased expression of the CD69 early activation marker), reduced proliferation (decreased Ki67 expression, decreased Stat5 phosphorylation), and decreased anti-tumor activity (decreased clearance of B16F10 tumor cells in a metastatic model) [146,148]. Importantly, NK cell hyporesponsiveness following repeated treatment with IL-2, IL-15 or IL-15 agonists was not linked to a decreased expression of the CD122 CD132 chains [144,146,148] indicating that NK cells should be able to respond to the cytokines when re-injected (Figure 3).

#### 4.1.2. In Macaques and Humans

This inability to increase NK cell activity in mice after multiple injections of cytokine was further supported by clinical trials in humans for IFNα, IL-12 and IL-15. For each of these cytokines, blood sampling at time points following each injection confirmed that NK cell activity or expansion at the second cycle of injection was increased but to a lesser extent when compared to the first cycle of injection [153,154,155,156,157]. In agreement with mice and human studies, NK cell hyporesponsiveness following cycles of injection with an IL-15 agonist was described in healthy and SIV+ macaques [158,159]. Furthermore, human NK cells treated continuously in vitro with IL-15 also present a decreased viability, a diminished signaling and a decreased function [160] confirming that repeated exposure to IL-15 is deleterious for NK cells.

### 4.2. Cytokine Treatments and Origin of NK Cell Hyporesponsiveness

The origin of this phenomenon stays ill-defined in mice as well as humans. In mice, one study hypothesized the implication of regulatory cells or immunosuppressive cytokines but lacked sufficient evidence [144]. Moreover, regulatory T cells, immunosuppressive cytokines such as IL-10 or TGFβ, and also IFNγ, were shown not to be involved in IL-15 repeated treatment [146,148]. Studies from Saito and colleagues in 1985 have shown that NK cell hyporesponsiveness following repeated treatment with cytokine was linked to a decreased percentage of large granular lymphocytes (LGL), the effector cell population responsible for NK cell-mediated cytotoxicity [142,143]. However, despite initial characterization of LGL as NK cells, LGL are now shown to contain T cells and NK cells, which therefore prevents us from supporting their hypothesis. The most recent studies are also not congruent with the origin of NK cell hyporesponsiveness. For Elpek et al., NK cell hyporesponsiveness arises after the continuous exposure to IL-15 which modifies the natural turnover of mature NK cells and leads to the generation of a large compartment of senescent or end-stage NK cells. They hypothesized that signaling downstream of IL-15 could be involved, revealing a NK cell-intrinsic mechanism leading to the hyporesponsive state [146]. On the contrary, we have shown that NK cell hyporesponsiveness was not a cell-intrinsic phenomenon, but was linked to the first injection of IL-15 that modified the environment surrounding NK cells and prevented them from responding to a second cycle of injection [148]. Lastly, the hyporesponsive status of NK cells following repeated injections with IL-12 has been linked to T cells, and demonstrated as direct elimination through a Fas-dependent mechanism of hepatic NK cells [145].

In humans, the origin of NK cell hyporesponsiveness is limited to hypothesis. For IL-12 repeated treatments, authors have discussed a putative protective feedback mechanism where IL-12 would induce IL-10 [145]. For IFNα, a direct antiproliferative effect of IFNα was suggested [154]. No hypothesis was formulated regarding IL-15 repeated treatments.

### 4.3. Recovery of NK Cell Hyporesponsiveness

The reversibility of this phenomenon is, furthermore, unclear. Indeed, only two studies pointed out that, in mice, after a first cycle of treatment with IL-2 or IFNα a second injection using a different cytokine restores NK cell responsiveness [143,144]. In contrast, after a first cycle of treatment with IL-15, a second cycle of treatment using a different cytokines does not restore NK cell responsiveness [146,148]. Additionally, increasing the resting period between two cycles of injection with IL-15 does not reverse NK cell hyporesponsiveness [148,159]. NK cell hyporesponsiveness driven by IL-15 injections seems to be restored after two cycles of treatment when CD44^+^ CD8 T cells are depleted at the time of the second stimulation [148].

In humans, recovery of NK cell responsiveness after multiple treatment with cytokines has not been studied. Obtaining new evidence for recovery of NK cell responsiveness would be beneficial for designing precise treatment schedules that prevent NK cell exhaustion.

## 5. Conclusions

In summary, NK cell hyporesponsiveness has been reported in different contexts. Indeed, this exhausted phenotype can arise during NK cell development, after short or continuous exposure to activating signals in the context of cancer or infection, but also after repeated treatments with cytokines involved in NK cell biology. Despite major scientific discoveries, the mechanism of NK cell regulation toward activation or hyporesponsiveness remains ill-defined. First, it is unclear which signals are necessary or sufficient for NK cells to become hyporesponsive. Then, whether NK cells need to experience a short or a prolonged exposure to activating factors is uncertain. Lastly, the reversibility of this NK cell hyporesponsiveness is vague. Indeed, while cytokines are under investigation to restore NK cell responsiveness in cancer or infection, they have also been shown to induce NK cell hyporesponsiveness after repeated treatment in vitro and in vivo. These results have to be taken into consideration in order to choose the appropriate strategy to enhance NK cell function. We could hypothesize that cytokines might be used to reverse NK cell hyporesponsiveness in cancer or infection, but their treatment-schedules should be carefully evaluated. In our immunotherapeutic era, where NK cells are considered as a major target in cancer immunotherapy, it is of importance to precisely understand the signals driving NK cell hyporesponsiveness. Decoding the required timing and the signals at stake to generate hyporesponsiveness is essential for the improvement of NK-cell based anti-tumor therapy.
